# SHP-2 Promotes the Maturation of Oligodendrocyte Precursor Cells Through Akt and ERK1/2 Signaling In Vitro

**DOI:** 10.1371/journal.pone.0021058

**Published:** 2011-06-20

**Authors:** Xiujie Liu, Yuanyuan Li, Yong Zhang, Yan Lu, Wei Guo, Peng Liu, Jiazhen Zhou, Zhenghua Xiang, Cheng He

**Affiliations:** Key Laboratory of Molecular Neurobiology, Institute of Neuroscience, Ministry of Education, Neuroscience Research Centre of Changzheng Hospital, Second Military Medical University, Shanghai, China; Medical College of Georgia, United States of America

## Abstract

**Background:**

Oligodendrocyte precursor cells (OPCs) differentiate into oligodendrocytes (OLs), which are responsible for myelination. Myelin is essential for saltatory nerve conduction in the vertebrate nervous system. However, the molecular mechanisms of maturation and myelination by oligodendrocytes remain elusive.

**Methods and Findings:**

In the present study, we showed that maturation of oligodendrocytes was attenuated by sodium orthovanadate (a comprehensive inhibitor of tyrosine phosphatases) and PTPi IV (a specific inhibitor of SHP-2). It is also found that SHP-2 was persistently expressed during maturation process of OPCs. Down-regulation of endogenous SHP-2 led to impairment of oligodendrocytes maturation and this effect was triiodo-L-thyronine (T3) dependent. Furthermore, over-expression of SHP-2 was shown to promote maturation of oligodendrocytes. Finally, it has been identified that SHP-2 was involved in activation of Akt and extracellular-regulated kinases 1 and 2 (ERK1/2) induced by T3 in oligodendrocytes.

**Conclusions:**

SHP-2 promotes oligodendrocytes maturation via Akt and ERK1/2 signaling in vitro.

## Introduction

Myelination in vertebrates has evolved to insulate axons and facilitate saltatory conduction of action potentials. Within the CNS, oligodendrocytes are responsible for the formation of myelin. Oligodendrocytes are derived from OPCs, which originate from the ventral of the ventricular zone. OPCs proliferate, migrate to, and spread over the CNS before differentiating into premyelinating oligodendrocytes [1], [2]. Subsequently, oligodendrocytes undergo morphological maturation and produce myelin components. Eventually, axons are myelinated through a complex process orchestrated by a series of extrinsic and intrinsic regulators [3].

Many growth factors, including neuregulins, insulin-like growth factor-I and ciliary neurotrophic factor, have been shown to regulate oligodendrocyte differentiation through activating their receptors expressed on the surface of oligodendrocytes [4], [5], [6]. Once these corresponding receptors are activated, the intracellular signals will be triggered mainly through a network of pathways regulated by the level of phosphorylation dependent on the opposing actions of protein kinases and protein phosphatases [7], [8]. It has been much documented that protein kinases were critical for oligodendrocyte differentiation [9], [10], [11], [12]. However the role of protein phosphatases in this process has yet to be further investigated.

SHP-2, a Src-homology 2 domain (SH2)-containing tyrosine phosphatase, is a widely expressed intracellular enzyme. SHP-2 has been shown to be involved in JAK/STATs, mitogen activated protein kinase (MAPK)/ERK1/2 and Phosphatidylinositol-3-kinase (PI3K)/Akt signaling cascade in various cell types [13]. It was also found to bind directly to a variety of receptor tyrosine kinases (RTKs) in response to stimulation by growth factors or cytokines [14], [15]. Recently, SHP-2 has been reported to play crucial roles in regulation of generation, proliferation and myelination of oligodendrocytes in vivo [16], [17]. However, the underlying mechanism remains to be clarified.

In the present study, we found that SHP-2 was persistently expressed during developmental process of oligodendrocytes. SHP-2 regulated the maturation of oligodendrocyte precursor cells via Akt and ERK1/2 signaling in vitro.

## Materials and Methods

### Animals and reagents

SD rats were obtained from Joint Ventures Sipper BK Experimental Animal (Shanghai, China). All animal experiments were undertaken in accordance with the National Institute of Health Guide for the Care and Use of Laboratory Animals, with the approval of Second Military Medical University Committee on Animal Care (permission No: SCXK-HU-2007-0003). SOV and BrdU were purchased from Sigma (St. Louis, MO). SHP-2 inhibitor (PTPi IV) was from Calbiochem (Darmstadt, Germany). Antibodies to NG2 and MBP were purchased from Millipore (Billerica, MA). Mouse monoclonal antibody to O4 was from Sigma. Rabbit polyclonal antibodies to SHP-2 were from Sant Cruz/Bioworld. Rabbit polyclonal antibodies to GFP were from Sant Cruz. Rabbit polyclonal antibodies against pERK, ERK, pAkt and Akt were from Cell Signaling. Antibody to GAPDH was from Kangchen. Mouse monoclonal anti-BrdU antibody was from Thermo and the In-Situ Cell Death Detection Kit, TMR red was from Roche.

### Primary cell culture

OPCs were isolated from SD postnatal day 1 rats as described previously [18]. Briefly, the forebrains were removed and diced into fragments in Hank's buffered salt solution (HBSS) and incubated at 37°C for 30 min with 0.125% trypsinase. Dissociated cells were plated on poly-L-lysine (PLL)-coated tissue culture flasks and grown at 37°C for 10 day in DMEM medium with 10% fetal calf serum (Gibco). OPCs were collected by shaking the flask overnight at 280 rpm at 37°C, resulting in 90% purity. For assessing maturation, OPCs were plated on cover slides in neurobasal medium supplemented with both B27 (2%) and conditional medium (CM) of B104 cells for 2–5 days and proliferated up to more than 20,000 cells per cm^2^. Then, OPCs were pre-treated with inhibitors before media were changed into the differentiation medium. Lentivirus-mediated ShRNA transfections were performed at least 3 days before differentiation induction. In maturation assay, 30 nM T3 was added to induce OPCs to differentiate into O4+ premyelinating oligodendrocytes after 1 day and into MBP+ cells after 3 days.

### Preparation of culture conditioned medium

Ninety percent confluent B104 cells were rinsed by incubation with Hank's balanced salt solution and cultured in neurobasal medium. Two days later, the conditioned medium was collected and filtered with 0.22 µm filters to remove cell debris. Media were used in OPCs amplification after dilution with the appropriate fresh medium at different concentration.

### Lentiviral vector construction and production

Lentivirus encoding shRNAs for SHP-2 were prepared by GenePharma (Shanghai, China). The shRNA sequence is as follows: 5′-GAUUCAGAACACUGGGGAC-3′, which was designed according to previous report [19]. For construction of lentiviral vector expressing SHP-2, human SHP-2 cDNA was amplified by PCR and subcloned to pWP vectors. To produce lentivirus containing SHP-2, HEK-293T cells were co-transfected with pWP-SHP2 plasmid and ViraPower Packaging Mix using Lipofectamine 2000 according to the manufacturer's guidelines.

### Western blot

Oligodendrocytes were rinsed briefly with ice-cold PBS and lysed for 5 min in sample buffer. The cell lysates were denatured by boiling for 10 minutes and then centrifuged for 10 min at 13,000 g at 4°C. Protein concentration was determined by the Bradford method. Proteins were separated by sodium dodecyl sulfate (SDS)-polyacrylamide gel and then transferred onto nitrocellulose membranes. Membranes were then blocked with 5% non-fat milk in 1XTBST(Tris 10 mM, NaCl 150 mM, Tween20 0.1%, pH 7.6) and incubated with primary antibodies. After incubating with horseradish peroxidase (HRP)-conjugated secondary antibodies (Kang Chen), immunoreactive bands were visualized by chemiluminescence reagents (ECL, Pierce).

### Immunohistochemistry and immunocytochemistry

Cultured cells were gently rinsed with PBS (10 mM sodium phosphate, pH 7.4, and 150 mM NaCl) and then fixed with 4% paraformaldehyde (PFA) for 20 min at room temperature. The fixed cells were incubated with primary antibodies overnight at 4°C and stained with corresponding secondary antibodies. For immunohistochemistry, animals were anesthetized and perfused intracardially with 4% PFA in 0.1 M phosphate buffer, pH 7.2. The brains were removed, post-fixed overnight in the same solution at 4°C, and then cryopreserved in 20% sucrose in 0.1 M phosphate buffer and embedded Twenty-micro-meter frozen sections were cut and incubated in blocking buffer containing 5% BSA and 0.5% TritonX-100 for 30 minutes and then incubated overnight at room temperature with the primary antibodies diluted in the same blocking buffer. After rinsing in PBS, sections were incubated with the appropriate secondary antibodies conjugated to fluorescein or rhodamine (Jackson ImmunoResearch and Vector Laboratories). Hoechst 33258 (10 µg/ml; Sigma) was used as nuclear marker. Images were obtained using the Nikon fluorescent microscope or a Leica SP2 confocal microscope.

### BrdU incorporation and TUNEL assays

Proliferation assay was manipulated as described previously [16]. Briefly, OPCs were purified and plated onto PLL-coated 24-well culture dishes. During transfection assay, the following supplements were added: B104 CDM or 30 nM T3. After 72 hours lentiviral-transfection, OPCs were incubated for 8 hours with 10 µM BrdU. During inhibitors assay, cultures were changed with the media supplemented with 30 nM T3 and 10 µM BrdU after OPCs were pre-treated with inhibitors. After culturing for 8 hours, the preparations were gently rinsed with PBS and fixed in 4% paraformaldehyde. Paraformaldehyde was removed 20 min later followed by three times of washing by PBS. Then, the preparations were treated with 2N HCL for 15 min, 0.1 M sodium borate, pH 8.5, for 15 min, and 0.2% Triton X-100 for 10 min at room temperature. Each step was followed by three times of washing by PBS. The preparations were successively performed according to immunocytochemical protocol. Percentages of BrdU-labeled cells against total or GFP-labeled cells were calculated. TUNEL assay was carried out using the in situ cell death detection kit (Roche) following manufacturer's instruction. In brief, the specimens were fixed in 4% PFA for 30 min and then processed for antigen retrieval with 0.1 M sodium citrate solution at room temperature. The specimens were then incubated with TUNEL reaction solution mixture containing terminal-deoxynucleo-tidyltra-nsferase (TdT) in a humidified chamber at 37°C for 30 minutes.

### RT-PCR

Total RNA was isolated using trizol reagents (Invitrogen) from cultures. The cDNA synthesis was performed using thermoscript RT-PCR system (Invitrogen). The following oligonucleotide primers were used: for rat SHP-2, the forward primer was 5′- GCAGTGC-TGGGATTGGCCGGACAGGAAC-3′, and the reverse primer was 5′- CAGTCAACCCCG-GCGAGCTTCTGAACAC-3′; for rat GAPDH, the forward primer was 5′-GCATGGCCTTC-CGTGTTCCTA-3′, and the reverse primer was 5′-AGTGTTGGGGGCTGAGTTGG-3′;

### Statistical analysis

All data are presented as mean ± SD. Statistical analysis was performed using ANOVA or unpaired Student's t-test. Statistical significance was calculated with *P* value of <0.05.

## Results

### PTPs inhibitors suppress oligodendrocyte maturation in vitro

**Figure 1:**
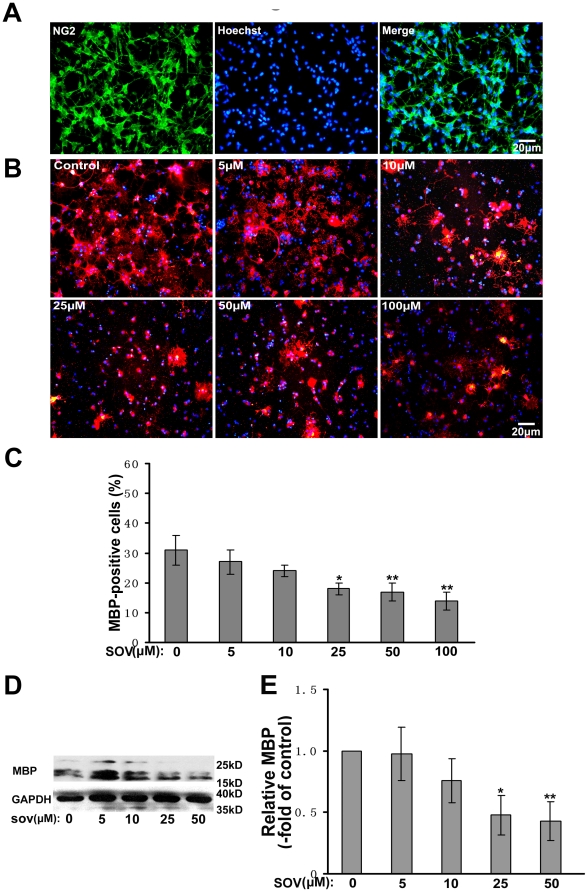
SOV inhibits expression of MBP in oligodendrocytes in vitro. (**A**) Identification of purified OPCs. (**B**) OPCs were pre-treated with different doses of SOV for 1 h every 24 h before the medium were changed to fresh differentiation medium without SOV. OPCs were immunostained with anti-MBP antibody (red) 3 days after maturation induction. The total number cells were identified by Hoechst staining (blue). The culture without SOV treatment was used as control. (**C**) Percentages of MBP-positive cells against the total number of cells. For each group, over 1000 cells were analyzed. *P<0.05, **P<0.01 vs controls. (**D**) After induction of maturation, OPCs treated with or without SOV were lysed and immunoblotted with antibody against MBP. GAPDH was used as a loading control. (**E**) Quantification of D was presented as fold of control. Results were from three independent experiments. *P<0.05, **P<0.01.

**Figure 2:**
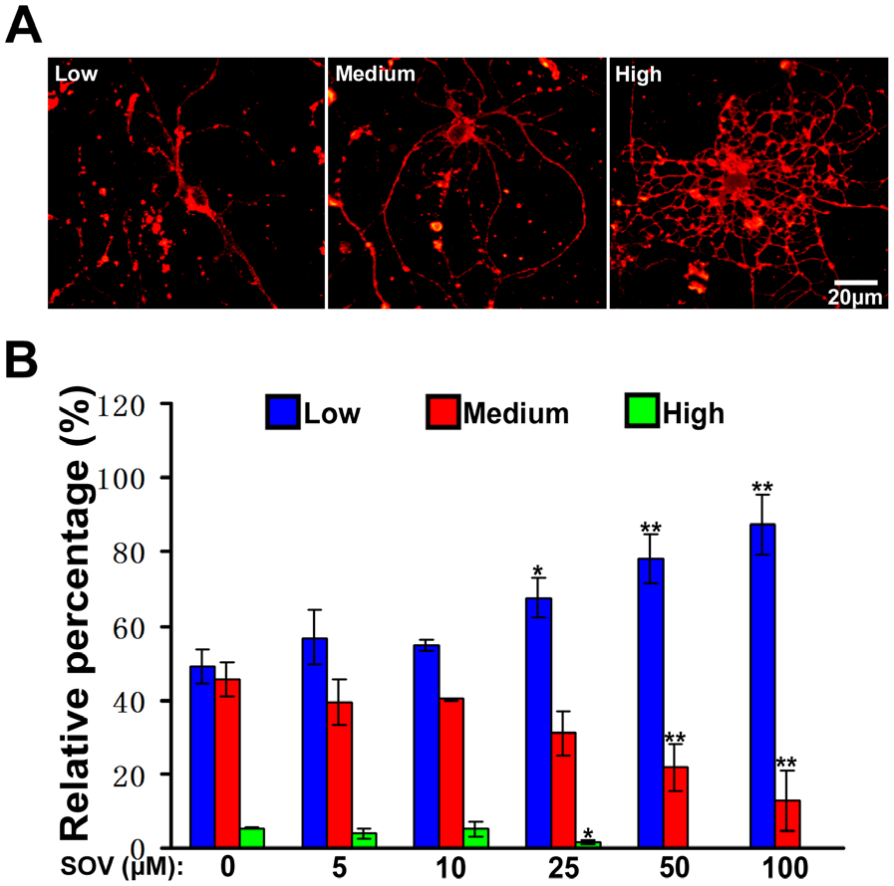
SOV inhibits morphological maturation of OPCs in vitro. (**A**) OPCs were pre-treated with different doses of SOV. After induction of maturation for 1 day, cells were fixed and stained with O4 monoclonal antibody. O4-positive cells were then categorized into three types. The representative images of cells in three categories are shown. (**B**) The percentages of the cells treated with or without SOV in each category were quantified after induction of maturation. For each group, over 1000 cells were analyzed. *P<0.05, **P<0.01 vs controls. Data represent five independent experiments.

Several tyrosine phosphatases have been found in the oligodendrocytes [20]. To investigate the effect of these phosphatases on oligodendrocyte maturation in vitro, we used SOV, a broad-spectrum inhibitor of tyrosine phosphatases [21], to interrupt activities of protein tyrosine phosphatases in the oligodendrocytes and detect their effect on oligodendrocyte maturation. OPCs were purified and cultured on poly-L-lysine-coated slides in neurobasal media supplemented with B27. The purity of OPCs was estimated to be over 95% using NG2 as a marker (Fig. 1A). The OPCs were induced to differentiate and pre-treated with SOV for 1 h every 24 h before fresh differentiation media were changed. The cells were immunostained with anti-MBP antibody 3 days after induction of maturation. The total number of cells was identified by Hoechst staining. The culture without SOV treatment was used as the control. We found that SOV treatment (≥25 µM) resulted in a significant decrease in the number of MBP imunopositive cells (Fig. 1B, C). Western blot analysis further showed that SOV treatment inhibited the expression of MBP in oligodendrocytes (Fig. 1D, E). Since maturation of oligodendrocytes involves dynamic morphological changes driven by cytoskeletal rearrangements, we wondered whether SOV participates in the morphological maturation of oligodendrocytes. The OPCs were cultured for 1 day in differentiation media with or without SOV, and immunostained by O4 subsequently. The representative morphology of maturation were categorized into three stages (Figure 2A) as described previously [22]. As shown in Fig. 2B, SOV treatment (≥25 µM) significantly inhibited maturation of oligodendrocytes into the stage with high morphological complexity of processes.

**Figure 3:**
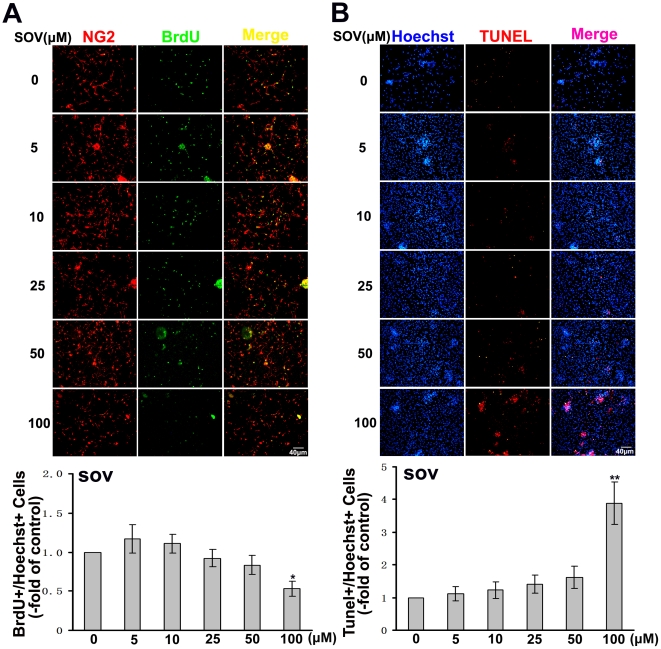
The effect of SOV on proliferation and apoptosis of the OPCs in vitro. (**A**) The primary cultures of OPCs were placed on PLL-coated dishes and cultured in different concentrations of SOV for 8 h with 10 µM BrdU. Proliferated cells were labeled with anti-BrdU (green), and cells were labeled with NG2 antibody (red). The culture without SOV was used as control and quantification was presented as fold of control. Ratio of BrdU-labeled cells was calculated. Data shown are the mean ± S.D. of three independent experiments. *P<0.05 vs controls. (**B**) Apoptotic cells were detected by TUNEL staining (red), and total number of cells was detected with Hoechst staining (blue). The culture without SOV was used as control and quantification was presented as fold of control. Ratio of TUNEL-labeled cells was calculated. Data shown are the mean ± S.D. of three independent experiments. **P<0.01 vs controls.

Next, we performed BrdU assay to evaluate the role of SOV on OPCs proliferation, Purified OPCs were cultured in different concentration of SOV for 8 h with BrdU. Proliferated cells were labeled with anti-BrdU and NG2 antibody. As shown in figure 3A, no significant difference in ratio of BrdU positive cells was found between low doses of SOV(≤50 µM) groups and the control group. However, proliferation of the 100 µM SOV group showed significantly decrease. We also performed TUNEL staining to evaluate the role of SOV in OPCs apoptosis. As shown in figure 3B, there was no significant difference in TUNEL-positive cells between low doses of SOV(≤50 µM) treated groups and the control except that apoptotic cells in the 100 µM SOV group showed significantly increase. Thus, high dose of SOV group (100 µM) inhibited both proliferation and maturation of the OPCs and induced apoptosis. We speculated that these may be due to the toxic effect of SOV on the cells.

**Figure 4:**
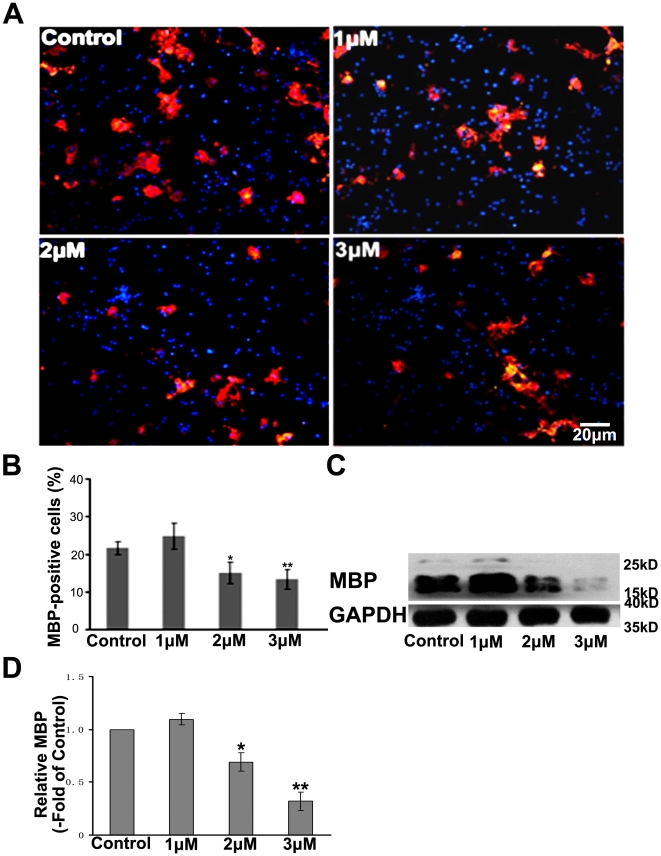
PTPi IV attenuates MBP expression of oligodendrocytes in vitro. (**A**) OPCs were pre-treated with different doses of PTPi IV for 6 h every 24 h before the medium were changed to fresh differentiation medium without PTPi IV. OPCs were immunostained with anti-MBP antibody (red) 3 days after induction of maturation. The total number of cells was identified by Hoechst staining (blue). The culture added DMSO was used as control. (**B**) Percentages of MBP-positive cells against the total number of cells. For each group, over 1000 cells were analyzed. *P<0.05, **P<0.01 vs controls. Data represent three independent experiments. (**C**) After induction of maturation, OPCs treated with different concentration of PTPi IV were lysed and immunoblotted with antibody against MBP with GAPDH as a loading control. (**D**) Quantification of C was presented as fold of Control. Results were from three independent experiments. *P<0.05, **P<0.01.

**Figure 5:**
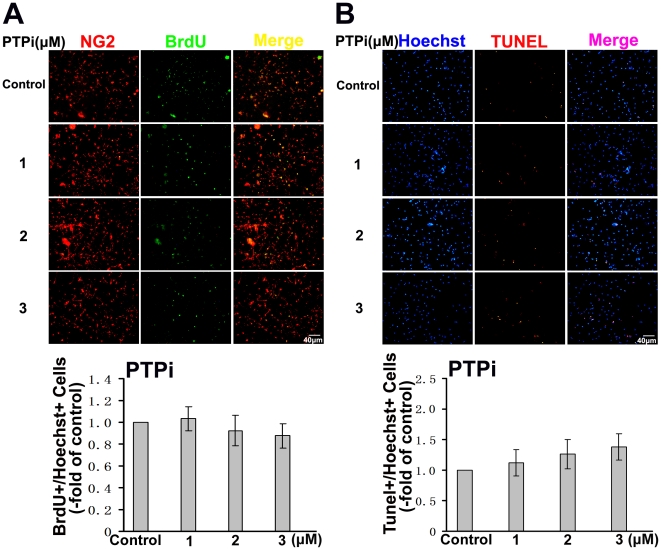
The effect of PTPi IV on proliferation and apoptosis of the OPCs in vitro. (**A**) The OPCs were cultured in different concentration of PTPi IV for 8 h with 10 µM BrdU. OPCs were labeled with NG2 antibody (red), and proliferated cells were labeled with anti-BrdU (green). The culture without PTPi IV was used as control and quantification was presented as fold of control. Ratio of BrdU-labeled cells was calculated. Data shown are the mean ± S.D. of three independent experiments. (**B**) Apoptotic cells were detected by TUNEL staining (red), and the total number of cells were identified by Hoechst staining (blue). The culture without PTPi IV was used as control and quantification was presented as fold of control. Ratio of TUNEL-labeled cells was calculated. Data shown are the mean ± S.D. of three independent experiments.

PTPi IV, protein tyrosine phosphatase Inhibitor IV, acts as a potent, reversible, competitive inhibitor of protein tyrosine phosphatases according to the product description of Calbiochem. Thus, we used PTPi IV to further identify the potential phosphatase affecting oligodendrocytes maturation. The cells were allowed to differentiate and PTPi IV was added for 6 h every 24 h as previous described [23] before the media were changed to fresh differentiation media without PTPi IV. The culture added DMSO was used as control. OPCs were immunostained with anti-MBP antibody 3 days after induction of maturation. The total OPCs were identified by Hoechst staining. The culture without PTPi IV treatment was used as the control. As shown in Fig. 4A and B, PTPi IV significantly decreased the number of MBP positive oligodendrocytes at the concentration of 2 µM. Western blot analysis further showed that PTPi IV treatment inhibited MBP expression in oligodendrocytes (Fig. 4C, D). We also examined the effect of PTPi IV on proliferation and apoptosis of OPCs, OPCs were cultured in different concentrations of PTPi IV for 8 h with BrdU. Proliferated cells were labeled with anti-BrdU and NG2 antibody. As shown in figure 5A, no difference in ratio of BrdU positive cells was found between PTPi IV groups and the control group in differentiation medium. We also performed TUNEL staining to evaluate the role of PTPi IV in OPCs apoptosis. As shown in figure 5B, there was no significant difference in TUNEL-positive cells after PTPi IV treatment at these concentrations. PTPi IV has relative specific inhibitory activity on SHP-2 at 2 µM concentration as previous described [23]. Thus, these data provide the clue that SHP-2 may affect the oligodendrocytes maturation without influencing proliferation and apoptosis in T3-supplemented differentiation media.

### SHP-2 is necessary for maturation of oligodendrocytes

**Figure 6:**
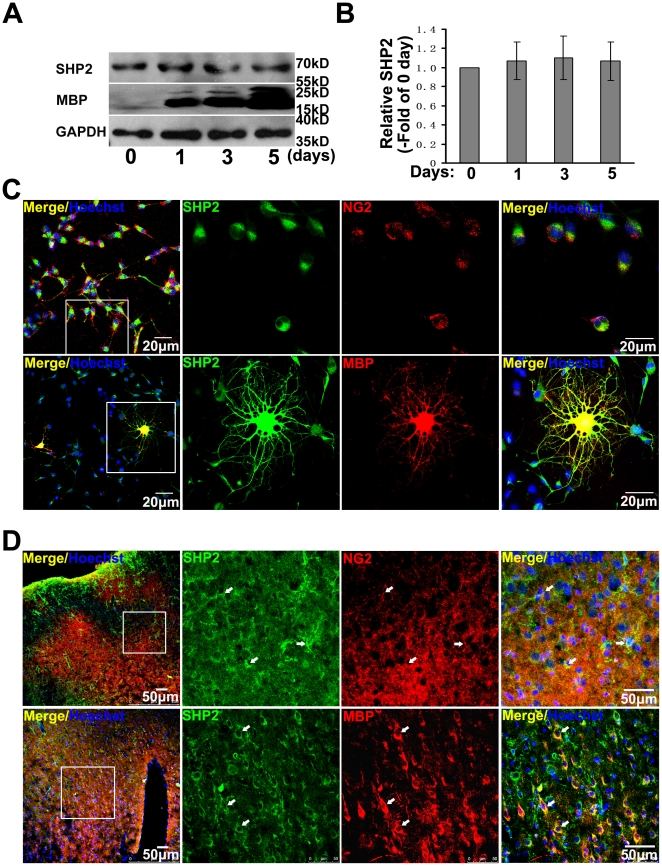
Expression of SHP-2 in oligodendrocytes in vitro and in vivo. (**A**) OPCs differentiated for 1, 3 and 5 days and analyzed protein levels of SHP-2 by western blot. GAPDH and MBP were used as control. (**B**) Densitometry to determine levels of SHP-2 protein, quantification presented as fold of control. Results were from three independent experiments. (**C**) Expression of SHP-2 in primary cultured rat OPCs. The distribution of SHP-2 (green) and NG2 (red) or MBP (red) is shown by immunofluorescence. (**D**) SHP-2 expression of oligodendrocytes in brain slices. The tissue slices from rat brain were immunostained with SHP-2 antibody (green) and markers of oligodendrocytes (red). Arrows indicate SHP-2 expression in oligodendrocytes.

To examine the role of SHP-2, we firstly detected the expression of SHP-2 in oligodendrocytes. OPCs were cultured and allowed to differentiate for 1, 3, or 5 days. Cell lysates were obtained and Western blot analysis using SHP-2 antibodies was performed. As shown in Fig. 6A and B, SHP-2 was persistently expressed during maturation process of OPCs. Double staining for SHP-2 and NG2 or MBP revealed that SHP-2 was expressed in both precursor and mature oligodendrocytes (Fig. 6C). To examine the expression patterns of SHP-2 in vivo, we carried out immunohistochemistry analysis in rat brain slices. As shown in Fig. 6D, in the cortex slices, both NG2 and MBP were observed to merge with SHP-2, suggesting that SHP-2 were expressed in oligodendrocytes in vitro and in vivo.

**Figure 7:**
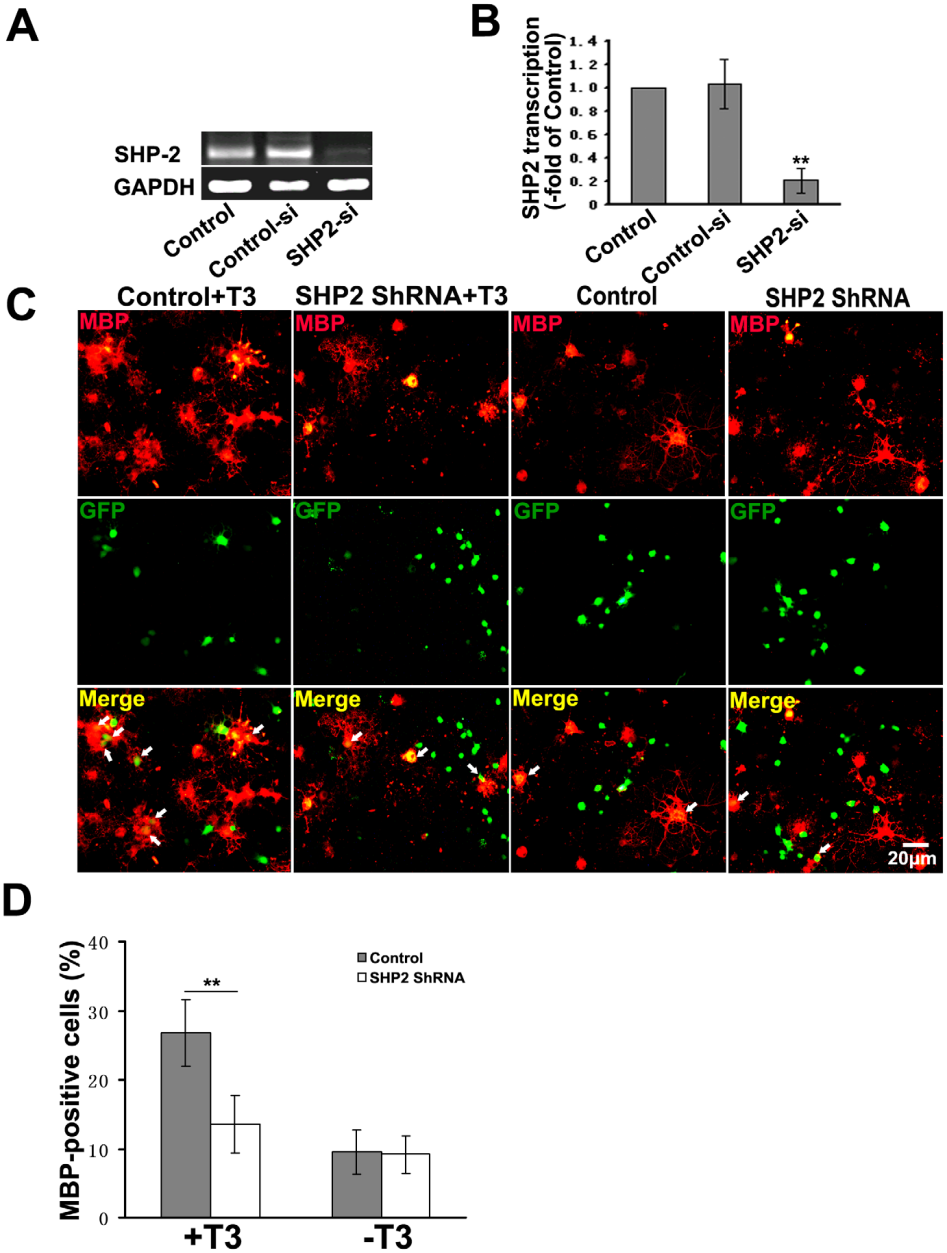
Knockdown of SHP-2 inhibits oligodendrocytes maturation. (**A**) RT-PCR analysis of SHP-2 mRNA transcription in OPCs infected with lentivirus expressing ShRNAs of SHP-2 (SHP2-si), Scramble (Control-si) or blank vector (Control). GAPDH was used as an internal standard. (**B**) Quantification of A, presented as fold of control. Results were from three independent experiments. **p<0.01. (**C**) OPCs infected with lentivirus expressing ShRNA were immunostained with anti-MBP antibody (red) after induction of maturation for 3 days. Arrows indicate MBP+ in GFP-expressing cells. The cells infected with Lentivirus expressing the control ShRNA and induced by T3 were used as control. (**D**) Percentages of MBP-positive cells (red) against the GFP-labeled cells, over 500 cells were analyzed for each group. Data represent three independent experiments. **p<0.01.

**Figure 8:**
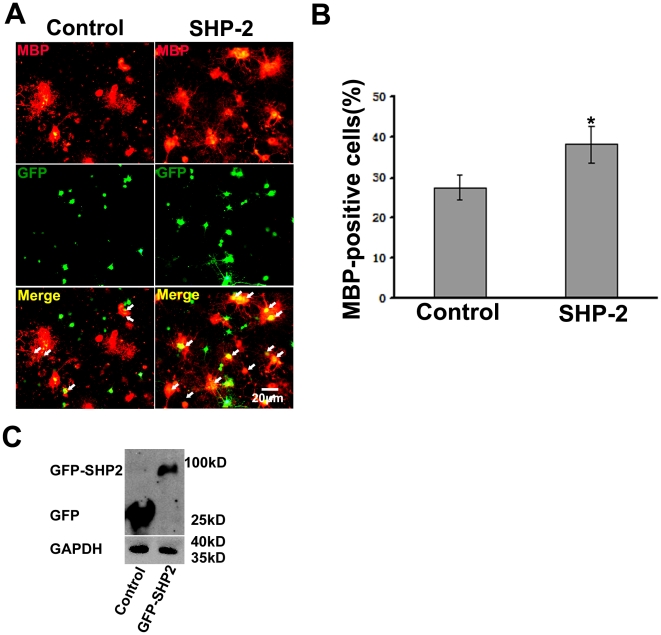
Overexpression of SHP-2 positively regulates oligodendrocyte maturation. (**A**) OPCs were infected with lentivirus expressing SHP-2. After induction of maturation for 3 days, cells were immunostained with anti-MBP antibody (red). Arrows indicate MBP+ in GFP-expressing cells. The cells infected with Lentivirus expressing the blank vector tagged by GFP were used as control. (**B**) Percentages of MBP-positive cells (red) against the total number of infected cells, over 500 cells were analyzed for each group. Data represent three independent experiments. *p<0.05. (**C**) The overexpression of GFP-SHP2 in transfected OPCs were detected by western blot using GFP antibody.

**Figure 9:**
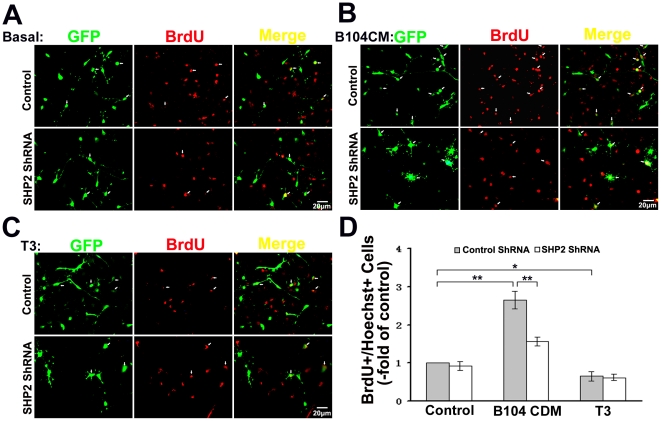
The effect of SHP-2 knockdown on proliferation of the OPCs in vitro. (**A, B and C**) OPCs were infected with the lentivirus expressing SHP-2 ShRNA and corresponding control. After 72 hours lentiviral-transfection, the medium was changed and the following supplements were added: B104 CDM (**B**) or 30 nM T3 (**C**). The basal medium (Neurobasal) was used as control (**A**). OPCs were incubated for 8 hours with 10 µM BrdU. Proliferated cells were labeled with anti-BrdU (red), and cells were labeled with GFP antibody (green). (**D**) Quantification of A was presented as -fold of control. The culture transfected with the blank vector was used as control. Ratio of BrdU-labeled cells was calculated. Data shown are the mean ± S.D. of three independent experiments. *P<0.05, **P<0.01.

To further confirm the specific effect of SHP-2 on OPCs maturation, we infected primary OPCs with a lentivirus expressing both GFP and a short-hairpin RNA (shRNA) sequence targeting SHP-2. The expression of SHP-2 was specifically knocked down by infection with this ShRNA, whereas the SHP-2 expression in the control was unaffected (Fig. 7A,B). Similar to the effect of PTPi IV, knockdown of SHP-2 reduced the number of MBP positive cells compared to those infected with the control ShRNA (Fig. 7C,D). Whereas, there was no significant difference in the number of MBP positive cells between SHP-2 knockdown ShRNA and the controls ShRNA without T3-induction (Fig. 7C,D). We also overexpressed SHP-2 in oligodendrocytes by lentivirus (Fig. 8C). In contrast to the effect of knockdown, overexpression of SHP-2 significantly increased the number of MBP positive cells in cultures supplemented with T3 (Fig. 8A,B). Next, we detected whether SHP-2 affect OPCs proliferation, The primary cultures of OPCs were placed on PLL-coated dishes and amplified in B104 CDM which is rich in mitogens. Then, the lentiviruses were added into the cultures. After 72 hours lentiviral-infection, OPCs were incubated for 8 hours with 10 µM BrdU and the following supplements were added into the media: B104 CDM or 30 nM T3. The neurobasal media was used as the control. Cells were labeled with anti-BrdU and GFP antibody. We observed that B104 CDM significantly increased OPCs proliferation compared to the control (Fig. 9A, B and D). T3 slightly inhibited OPCs proliferation (Fig. 9A, C and D). After SHP-2 knockdown, OPCs proliferation significantly decreased in the culture supplemented with B104 CDM (Fig. 9B and D). However, no difference in ratio of BrdU positive cells was observed in the basal medium group and the cells supplemented with T3 (Fig. 9A, C and D). These data indicate that SHP-2 is required not only for OPCs proliferation by mitogens stimulation but also for maturation induced by T3.

### Akt and ERK1/2 signaling are involved in mediating the effect of SHP-2 on oligodendrocyte maturation

**Figure 10:**
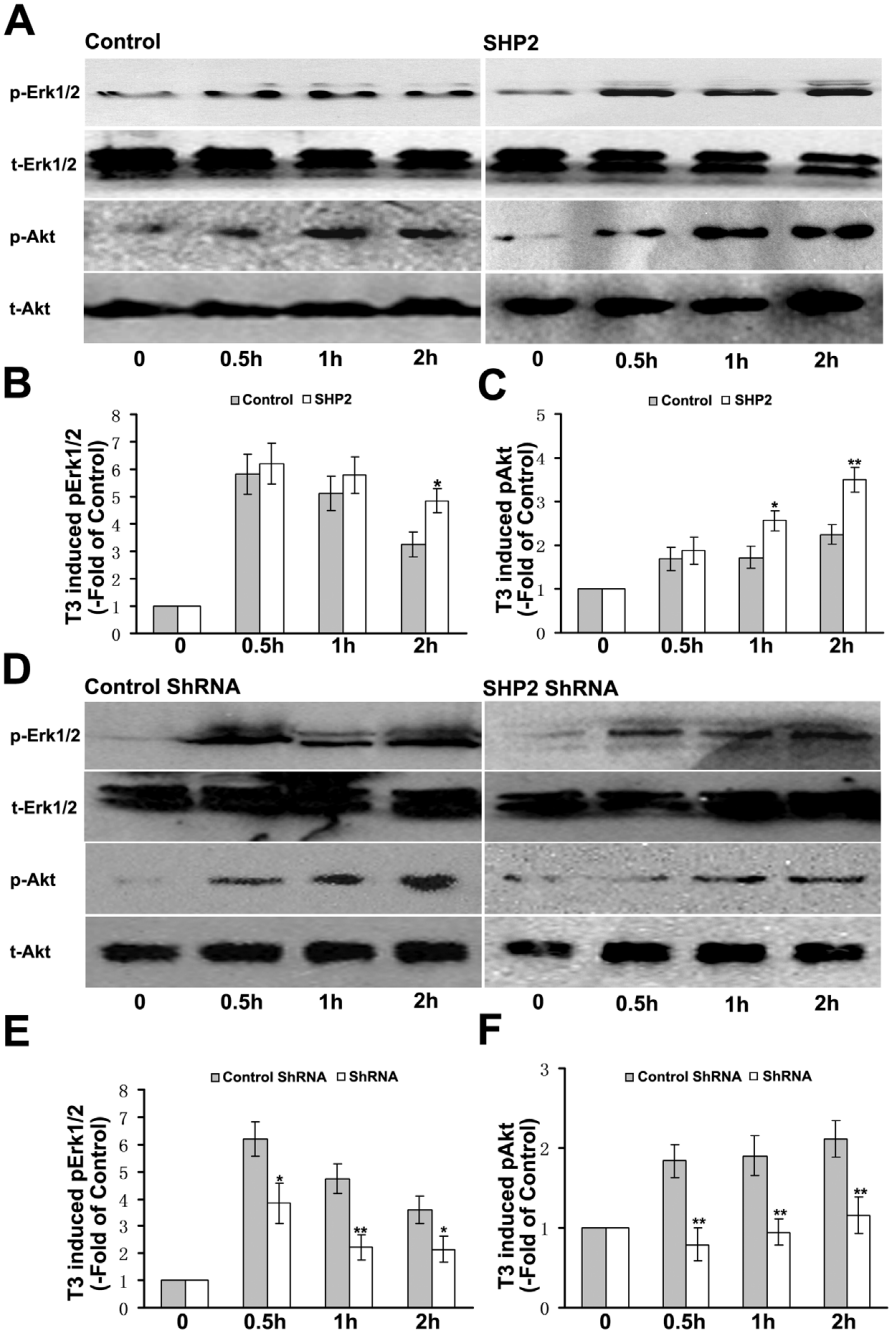
SHP-2 is involved in T3-induced activation of Akt and ERK1/2 in OPCs. (**A**) OPCs were infected with lentivirus expressing SHP-2 using blank vector as control. Activation of Akt and ERK1/2 in oligodendrocytes was assessed with western blot after T3 stimulation in transfected OPCs. (**B, C**) Quantification of A with densitometric analysis was presented as fold of control. Results were from three independent experiments. *p<0.05 and **p<0.01. (**D**) Activation of Akt and ERK1/2 in oligodendrocytes were assessed with western blot after T3 stimulation in OPCs transfected with SHP-2 ShRNA or blank vector. (**E, F**) Quantification of D with densitometric analysis was presented as fold of control. Results were from three independent experiments. *p<0.05 and **p<0.01.

**Figure 11:**
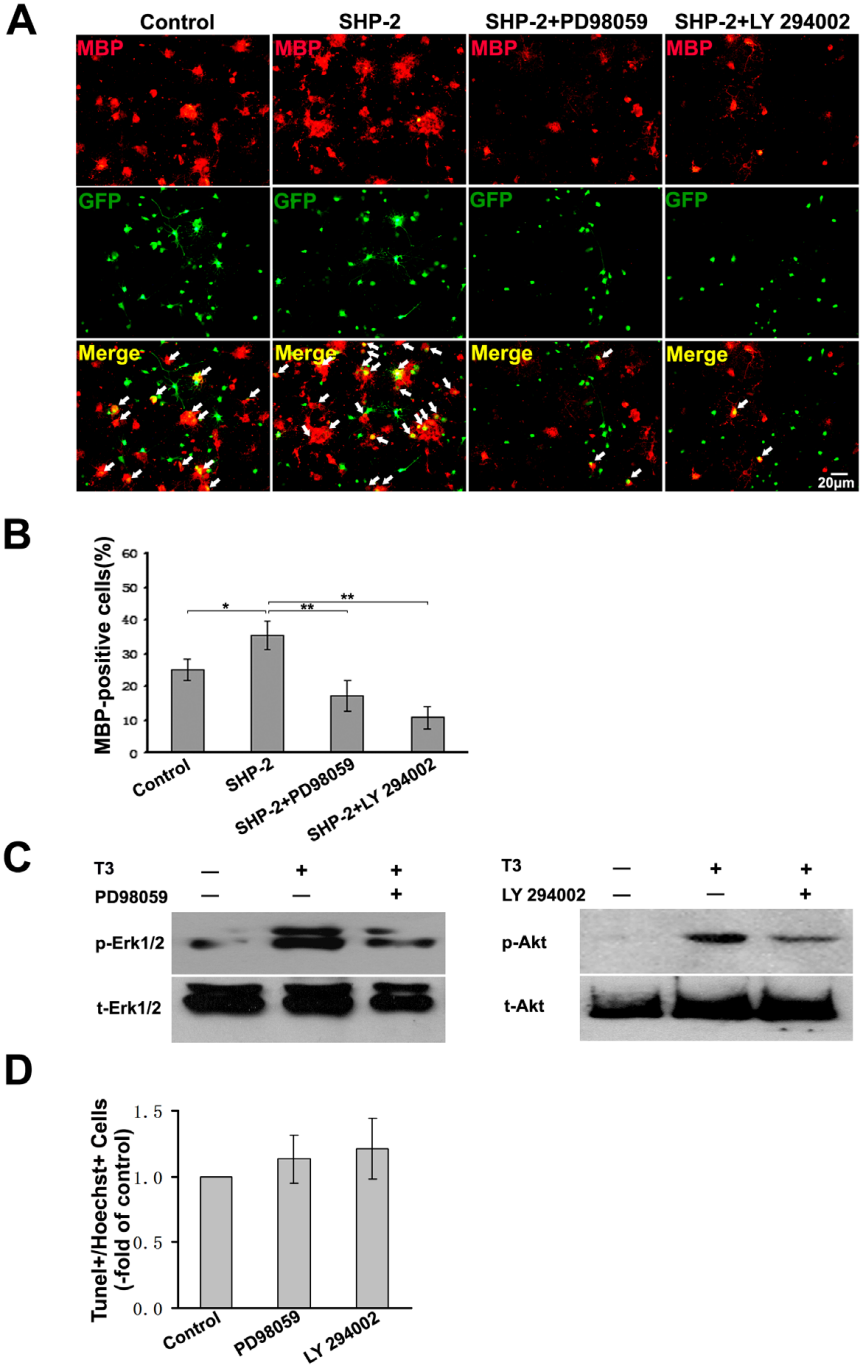
Inhibition activation of Akt and ERK1/2 attenuates the promotional effect of SHP-2 on oligodendrocyte maturation. (**A**) OPCs were infected with lentivirus expressing SHP-2 using blank vector as control. Cultures transfected with SHP-2 were pre-treated with 2.0 µM PD98059 (MEK1/2 inhibitor) or 20 µM LY294002 (PI3K inhibitor) for 30 min, respectively. Cells were immunostained with anti-MBP antibody (red). Arrows indicate MBP+ in GFP-expressing cells. The cells infected with Lentivirus expressing the blank vector tagged by GFP were used as control. (**B**) Percentages of MBP-positive cells (red) against the number of infected cells. Over 500 cells were analyzed for each group. Data represent three independent experiments. *p<0.05 and **p<0.01. (**C**) Activation of Akt and ERK1/2 in oligodendrocytes induced by T3 (30 nM) were assessed with western blot after inhibitors pre-treatment. (**D**) Pre-treatment with PD98059 (2.0 µM) or LY294002 (20 µM) did not induce significant OPCs death in cell culture system.

Activation of the Akt and ERK1/2 are known to be crucial for the differentiation of OPCs [8], [24] and T3 has been shown to induce the activation of Akt and ERK1/2 in human fibroblasts and myoblasts [25], [26], [27]. To demonstrate that SHP-2 was involved in regulating oligodendrocytes maturation through Akt and ERK1/2 signaling, we detected the activation of Akt and ERK1/2 after gene-manipulation of SHP-2 expression in OPCs. As shown in Figure 10A, B and C, Western blot analysis revealed that OPCs supplemented with T3 resulted in prominent activation of both ERK1/2 and Akt, and SHP-2 overexpression increased the T3-induced activation of Akt and ERK1/2 in the OPCs. It seemed that the promotional effect of SHP-2 overexpression on the activation of Akt is more significant than that on ERK1/2. Meanwhile, after knockdown the expression of SHP-2, these activations were significantly inhibited (Fig. 10D, E and F). To further characterize the signaling pathways mediating the effect of SHP-2, we examined whether these signaling pathways were involved in T3–induced oligodendrocytes maturation. The cultured cells were pre-treated with specific inhibitors of various protein kinases for 30 min as previous described [28]. After pre-treatment, the activation of Akt and ERK1/2 by T3 were significantly reduced in the OPCs without affecting cells survival (Fig. 11C and D). As shown in Figure 11A and B, the promoting-effect of SHP-2 overexpression on oligodendrocyte maturation was eliminated by pre-treatment with LY 294002, a selective PI3K inhibitor. Meanwhile, PD98059, a selective inhibitor of MAPK, also attenuated the acceleration of oligodendrocytes maturation by SHP-2 overexpression. Thus, our data supported the notion that SHP-2 may be involved in regulating T3-induced oligodendrocytes maturation via Akt and ERK1/2 signaling.

## Discussion

Several studies have been reported that protein phosphatases exist in oligodendrocytes and function as a regulator for differentiation and maturation of oligodendrocytes [29], [30]. Genetic deletions of SHP-1, the phosphatase and tensin homolog deleted on chromosome 10 (PTEN) and episilon have been shown to negatively regulate myelination [31], [32], [33]. Recently, receptor-like protein tyrosine phosphatase α (PTPα) has been shown to promote oligodendrocyte differentiation and myelination through regulating Fyn signaling [29]. Furthermore, Zhu et al reported that maturation and myelination of oligodendrocytes were compromised in SHP-2 conditional mutants [17]. In the present study, we firstly used SOV to examine the effect of comprehensive inhibition of tyrosine phosphatases on the development of OPCs. We found that SOV significantly inhibited the maturation of OPCs in vitro. Moreover, we found that the maturation of oligodendrocytes was significantly hindered by PTPi IV treatment. These results were confirmed by SHP-2 knockdown. Our data provided evidences that SHP-2 positively regulates oligodendrocytes maturation. It has been reported that SHP-2 regulates the phosphatidylinositide 3′-kinase/Akt pathway and suppresses caspase 3-mediated apoptosis in fibroblasts and myoblasts [34]. However, no significant apoptosis increase was observed 3 days after induction of maturation in PTPi IV-treated OPCs. We speculated that this may be due to that T3 promotes survival of OPCs in vitro.

Kuo et al. reported that tyrosine phosphatases SHP-1 and SHP-2 had unique and opposing roles in oligodendrocyte development. They found that SHP-2 depletion did not prevent oligodendrocyte differentiation in vitro [16], which is inconsistent with our results. It is likely that the differences of the culture system may account for these conflicting results. In our study, T3 was added into the differentiation media to induce oligodendrocytes maturation. We found that there was no significant effect on oligodendrocytes maturation when SHP-2 was knocked down without T3 induction, suggesting that SHP-2 may play a key role in T3-induced OPCs maturation.

SHP-2 has been shown to be involved in a number of signaling pathways. It has been reported that the activation of Ras/Raf/ERKs signaling pathway is positively regulated by SHP-2 [35]. Additionally, SHP-2 regulates growth factor-mediated PI3K/Akt pathway. Ectopic expression of SHP-2 in U87MG gliobastoma cells elevated EGF-induced Akt phosphorylation. Deletion of SHP-2 in mouse fibroblasts reduced phosphorylation of Akt and ERK1/2 [34], [36]. Both Akt and ERK1/2 signaling have been demonstrated to play key roles in differentiation of oligodendrocytes [8], [24]. In this study, we found that the phosphorylation levels of Akt and ERK1/2 in T3-induced differentiating oligodendrocytes were greatly increased. More importantly, the promotional effect of SHP-2 overexpression on oligodendrocyte maturation was eliminated by treatment with specific inhibitors of Akt and ERK1/2, indicating the involvement of these signaling pathways in maturation of oligodendrocytes. Thyroid hormone has been well documented to be a key regulator in oligodendrocytes by triggering the onset of differentiation [37], [38], [39]. Moreover, cytosolic thyroid hormone receptors can interact with the p85α regulatory subunit of PI3K and activate PI3K/Akt [26]. T3 can also induce activation of MAPK/ERK via putative G-protein-coupled receptor [27]. In our study, SHP-2 knockdown in oligodendrocytes was found to significantly down-regulate the activation of ERK1/2 and Akt induced by T3. Conversely, SHP-2 overexpression increased the T3-induced activation of Akt and ERK1/2 in the OPCs. Our data strongly suggested that SHP-2 may function at the downstream of T3 signaling and promote oligodendrocytes maturation through facilitating activation of Akt and ERK1/2 signaling. However, the detail molecular mechanism underlying SHP-2 involved in this process should be further detected.

Purified population of OPCs have an intrinsic timing mechanism that controls the number of cell divisions before entering cell differentiation, while disruption of mitogenic signaling causes OPCs to exit cell cycle and differentiate prematurely. Recent study reported that SHP-2 knockdown decreased OPCs proliferation [16]. Thus, it is important to determine whether the reduction in the number of MBP+ cells by SHP-2 ShRNA is secondary to cell cycle defects. In our proliferation assay, we observed that SHP-2 knockdown significantly decreased OPCs proliferation in the culture supplemented with B104 CDM which is rich in mitogens such as platelet-derived growth factor (PDGF) and et al [40]. However, no difference in ratio of BrdU positive cells was observed in both the basal and T3-supplemented media system. Similarly, we also found no significant changes of OPCs proliferation after PTPi IV inhibitor treatment in T3-supplemented differentiation system. OPCs proliferation showed slightly decrease after T3 treatment which is consistent with the previous report [38]. Thus, we speculate that SHP-2 may play various roles in OPCs development and these effects were affected by the distinct extracellular stimulations. The development of oligodendrocytes in vivo results from sequential series of events including proliferation, migration, differentiation, myelination and et al. Each of these processes is regulated by various signals [3]. Our results showed that SHP-2 was persistently expressed in oligodendrocytes from early stage to terminal differentiation. These data indicated that SHP-2 is required not only for OPCs proliferation by mitogens stimulation but also for maturation induced by promotional differentiation factors.
